# Supplementary material for: Parental non-involvement strategy for handling sibling conflict on social avoidance in migrant children: Chain mediation of sibling conflict and parent-child conflict
Source: PLoS One. 2024 Sep 10;19(9):e0308561. doi: 10.1371/journal.pone.0308561 (PMC11386452; doi:10.1371/journal.pone.0308561)
Supplement: S1 Appendix — (DOCX) [file pone.0308561.s002.docx]

**Appendix A**

Constructs, Measurement Items and Sources

| **Constructs** | **No.** | **Measurement Items** | **Sources** |
| --- | --- | --- | --- |
| **Non-involvement Strategy** | NS1 | When I see kids arguing or fighting, I don't intervene. | (Jia lun Zhang., 2010) |
|  | NS2 | When he gets into an argument with his siblings, I just say stop it! And go on about my business without asking them what's going on. |  |
|  | NS3 | When he argued or fought with his siblings, I would ignore their conflicts because I was busy with something at hand. |  |
|  | NS4 | When one of my kids comes in to tattle, I just say, “Well, I know!” And I don't deal with their arguments. |  |
|  | NS5 | When he and his siblings fight over watching different cartoon shows, I just say don't even watch them! And ignore their fights. |  |
|  | NS6 | When a child argues or fights, I just tell the child:Neither can play! and ignore the argument. |  |
| **sibling conflict** | SC1 | He and his siblings would yell at each other. | (Furman & Buhrmeste., 1985) |
|  | SC2 | In many things, he and his siblings want to outdo or beat each other (want to perform better than each other). |  |
|  | SC3 | He and his siblings argue with each other when there are disagreements between them. |  |
|  | SC4 | He and his siblings want to do better than the other. |  |
|  | SC5 | He fights a lot with his siblings. |  |
| **Parent-child Conflict** | P-CC1 | He and I always seem to be fighting against each other. | (Pianta & Virginia., 2011) |
|  | P-CC2 | He gets angry with me easily. |  |
|  | P-CC3 | He sees me as the source of his punishment and criticism. |  |
|  | P-CC4 | He shows sadness or jealousy when I'm around other kids. |  |
|  | P-CC5 | He will remain angry or resistant after being punished. |  |
|  | P-CC6 | Spending time with him drained me. |  |
|  | P-CC7 | When he's in a bad mood, I know it's going to be a long, hard day for both of us. |  |
|  | P-CC8 | His moods towards me would suddenly change or elude me. |  |
|  | P-CC9 | Despite my best efforts, I felt awkward about my time with him. |  |
|  | P-CC10 | He grunts or cries when he wants something from me. |  |
|  | P-CC11 | He'll play tricks on me or manipulate me. |  |
| **Social Avoidance** | SA1 | He doesn't want to play with the other kids. | (Sang, B., Ding, X., Coplan, R. J., Liu, J., Pan, T., & Feng, X., 2018) |
|  | SA2 | When other children play games, he tends to watch rather than join in. |  |
|  | SA3 | He often refused social invitations from other children because he wanted to be left alone. |  |
|  | SA4 | He's shy when it comes to meeting new people for the first time. |  |
