# Supplementary material for: Parental non-involvement strategy for handling sibling conflict on social avoidance in migrant children: Chain mediation of sibling conflict and parent-child conflict
Source: PLoS One. 2024 Sep 10;19(9):e0308561. doi: 10.1371/journal.pone.0308561 (PMC11386452; doi:10.1371/journal.pone.0308561)
Supplement: Attachment — Submitted filename: recm.docx [file pone.0308561.s003.docx]

• **Abstract Clarity**: The abstract provides a good overview but can be improved for clarity. Consider explicitly stating the research design, key findings, and implications in a structured manner (e.g., Objectives, Methods, Results, Conclusions).

• **Literature Review**: The introduction could benefit from a more comprehensive literature review. Incorporate recent studies on sibling conflict, parental non-involvement, and social avoidance to establish a stronger theoretical foundation.

• **Hypotheses Statement**: Clearly state your research hypotheses at the end of the introduction. This helps in guiding the reader and setting clear expectations for the study.

• **Sample Size Justification**: Provide a justification for the sample size of 253 children. Include a power analysis to demonstrate that the sample size is adequate to detect the expected effects.

• **Measurement Tools**: Provide more details on the psychometric properties of the measurement tools used (e.g., validity, reliability). Mention any cultural adaptations made for the scales used in the Chinese context.

• **Data Collection Method**: Detail the data collection process, including how participants were recruited and how the surveys were administered. Mention any ethical considerations and how informed consent was obtained.

• **Statistical Analysis**: Explain the choice of statistical methods in more detail. For example, discuss why the SPSS macro developed by Hayes was chosen for mediation analysis and how the results were interpreted.

• **Common Method Bias**: The study mentions using Harman's one-way test for common method bias. Consider discussing the potential limitations of this test and explore additional methods to mitigate common method bias.

• **Discussion Depth**: The discussion section should be more comprehensive. Relate your findings to existing literature, explain possible reasons for your findings, and discuss the practical implications for parents and educators.

• **Limitations and Future Research**: While some limitations are mentioned, provide a more thorough discussion. Suggest specific areas for future research, such as longitudinal studies or the impact of different parental strategies on sibling conflict resolution.

• **Figures and Tables**: Ensure that all figures and tables are clearly labeled and provide additional context where necessary. For example, Figure 1 should be referenced in the text with a brief explanation of its contents.

• **References**: Update the references section to include the most recent studies and ensure all citations are properly formatted according to the journal's guidelines.

• **Ethical Considerations**: Expand on the ethical considerations in the methodology section. Mention the ethical review board's approval and any measures taken to protect participants' confidentiality and well-being.

• **Language and Style**: Ensure the manuscript is free from grammatical errors and uses clear, concise language. Consider professional editing to enhance readability.

• **Supplementary Materials**: If applicable, provide supplementary materials such as survey questionnaires or detailed statistical outputs to support the findings.
